# A Prospective, Open‐Label, Post Marketing Study of the Safety and Effectiveness of a Hyaluronic Acid and Calcium Hydroxyapatite Hybrid Injectable

**DOI:** 10.1111/jocd.70430

**Published:** 2025-09-15

**Authors:** Alessandro Gritti, Malka Salomon, Lea Elmaleh, Graeme Kerson, Andrew Schumacher

**Affiliations:** ^1^ Dr. Alessandro Gritti Oral and Maxillofacial Surgery Brescia Italy; ^2^ Allergan Aesthetics, an AbbVie Company Lod Israel; ^3^ Allergan Aesthetics, an AbbVie Company Marlow UK; ^4^ Allergan Aesthetics, an AbbVie Company Irvine California USA

**Keywords:** biostimulatory, CaHA, calcium hydroxyapatite, dermal filler, facial aging, HArmonyCA, hyaluronic acid

## Abstract

**Background:**

HA‐CaHA is a hybrid injectable combining hyaluronic acid and calcium hydroxyapatite for dual‐mode facial soft tissue augmentation. HA‐CaHA‐L includes 0.3% lidocaine HCL.

**Objective:**

This prospective, open‐label study evaluated the effectiveness and safety of HA‐CaHA for treating midface volume deficit.

**Methods:**

Adults rated Moderate to Severe on the Allergan Midface Volume Deficit Scale (MFVDS) received HA‐CaHA (*n* = 110) or HA‐CaHA‐L (*n* = 30) in the cheek (primary injection region) and jawline (optional) areas, with optional touch‐up 2 weeks later. Follow‐up visits occurred at Months 1, 3, 6, 9, and 12. The primary endpoint was the MFVDS responder rate (≥ 1‐grade improvement from baseline) at Month 1. Secondary endpoints were the Global Aesthetic Improvement Scale (GAIS) responder rates and change from baseline on FACE‐Q Satisfaction with Cheeks and Satisfaction with Facial Appearance. Exploratory endpoints were the Allergan Cheek Smoothness Scale (ACSS) scores and 3D photo‐based midface movement tracking. Safety was assessed throughout.

**Results:**

At Month 1, MFVDS responder rate was 82.8%, remaining high through Month 12. The GAIS responder rates were 98.5% (investigator‐rated) and 91.8% (participant‐rated) at Month 1, remaining high through Month 12. FACE‐Q scores improved 33.3 (Cheeks) and 23.8 (Facial Appearance) points at Month 1, remaining high through Month 12. ACSS scores peaked at Months 3 (73.7%) and 6 (69.2%), with corresponding skin movement changes. Most participants (95.7%) experienced mild or moderate ISRs. The most common treatment‐related AEs were injection site pain (11.4%), injection site mass (10.7%), and headache (7.1%).

**Conclusions:**

HA‐CaHA and HA‐CaHA‐L are effective and well‐tolerated for midface soft tissue augmentation, lasting up to 1 year.

## 1 Introduction

Facial aging has a complex and multifaceted etiology involving both intrinsic (e.g., genetics, hormonal changes) and extrinsic (e.g., UV irradiation/photoaging, smoking) factors [1]. As individuals age, structural and functional changes in skin and the underlying soft tissue and bone result in facial volume loss and sagging, contributing to the appearance of aging [2, 3]. Dissatisfaction with facial appearance can decrease an individual's self‐esteem and have negative psychological impacts that detract from their quality of life. Accordingly, there is growing interest in aesthetic products and procedures that provide facial volumizing and rejuvenation [4].

Injection with dermal fillers, which often contain either hyaluronic acid (HA) or calcium hydroxyapatite (CaHA), can rapidly augment soft tissue to enhance skin quality and restore facial volume loss. Whereas HA‐based dermal fillers provide rapid volumizing and lift, CaHA‐based fillers stimulate neocollagenesis, resulting in a longer‐lasting lifting effect and improvements in skin quality and laxity [5, 6, 7]. HA‐CaHA (HArmonyCa; Allergan Aesthetics, an AbbVie company; Irvine, CA) is a hybrid soft tissue filler that combines a crosslinked HA (20 mg/mL) gel matrix with CaHA microspheres (55.7% w/w) in a single ready‐to‐use syringe. The lidocaine version (HA‐CaHA‐L) includes 0.3% (w/v) lidocaine HCL. HA‐CaHA confers the advantages of its constituent components, with the HA providing immediate volume and lift, while the collagen‐stimulating effect of the CaHA promotes dermal thickening and improved skin quality, as well as a sustained lifting effect [8].

A few recent studies have demonstrated patient satisfaction, effectiveness, and a favorable safety profile associated with HA‐CaHA for midface soft tissue augmentation. One prospective study with 15 participants found that a single treatment with HA‐CaHA in the subzygomatic and posterior zygomatic regions produced sustained aesthetic improvements, as measured by the Global Aesthetic Improvement Scale (GAIS), that lasted for up to 120 days [9]. Likewise, another prospective study of 15 participants noted sustained improvements in midface volume and skin viscoelasticity following treatment with HA‐CaHA‐L that lasted through 6 months [10]. In each of these prospective studies, as well as a recent retrospective analysis that examined the safety of HA‐CaHA in over 400 patients [11], HA‐CaHA was found to be well tolerated, with only a few reports of mild or moderate treatment‐related adverse events and no reports of serious treatment‐related adverse events.

To expand on these findings, the present open‐label, post‐marketing study collected prospective data on the safety and effectiveness of HA‐CaHA and HA‐CaHA‐L for midface soft tissue augmentation.

## 2 Methods

### 2.1 Study Design

This 12‐month, open‐label, prospective study was conducted at 2 sites in France between October 2021 and September 2023. The study consisted of an initial treatment plus an optional touch‐up treatment 14 days later. Safety phone calls were made 3 days after any treatment. In‐person evaluations occurred at Months 1, 3, 6, 9, and 12 after initial treatment.

This study was conducted in accordance with the protocol, ICH guidelines, ISO 14155 medical device guidelines, applicable regulations and guidelines governing clinical study conduct, and ethical principles that have their origin in the Declaration of Helsinki. All participants provided informed written consent.

### 2.2 Participants

Eligible participants were adults with investigator‐rated midface volume deficit of 3, 4, or 5 on the validated Allergan Midface Volume Deficit Scale (MFVDS; Grade 1 = Minimal; Grade 2 = Mild; grade 3 = Moderate; Grade 4 = Significant; Grade 5 = Severe) at baseline [12, 13]. Participants were excluded from the study for known contraindications associated with HA‐CaHA or HA‐CaHA‐L, facial trauma within 6 months of study start date, or any prior confounding aesthetic treatments.

### 2.3 Treatments

**FIGURE 1:**
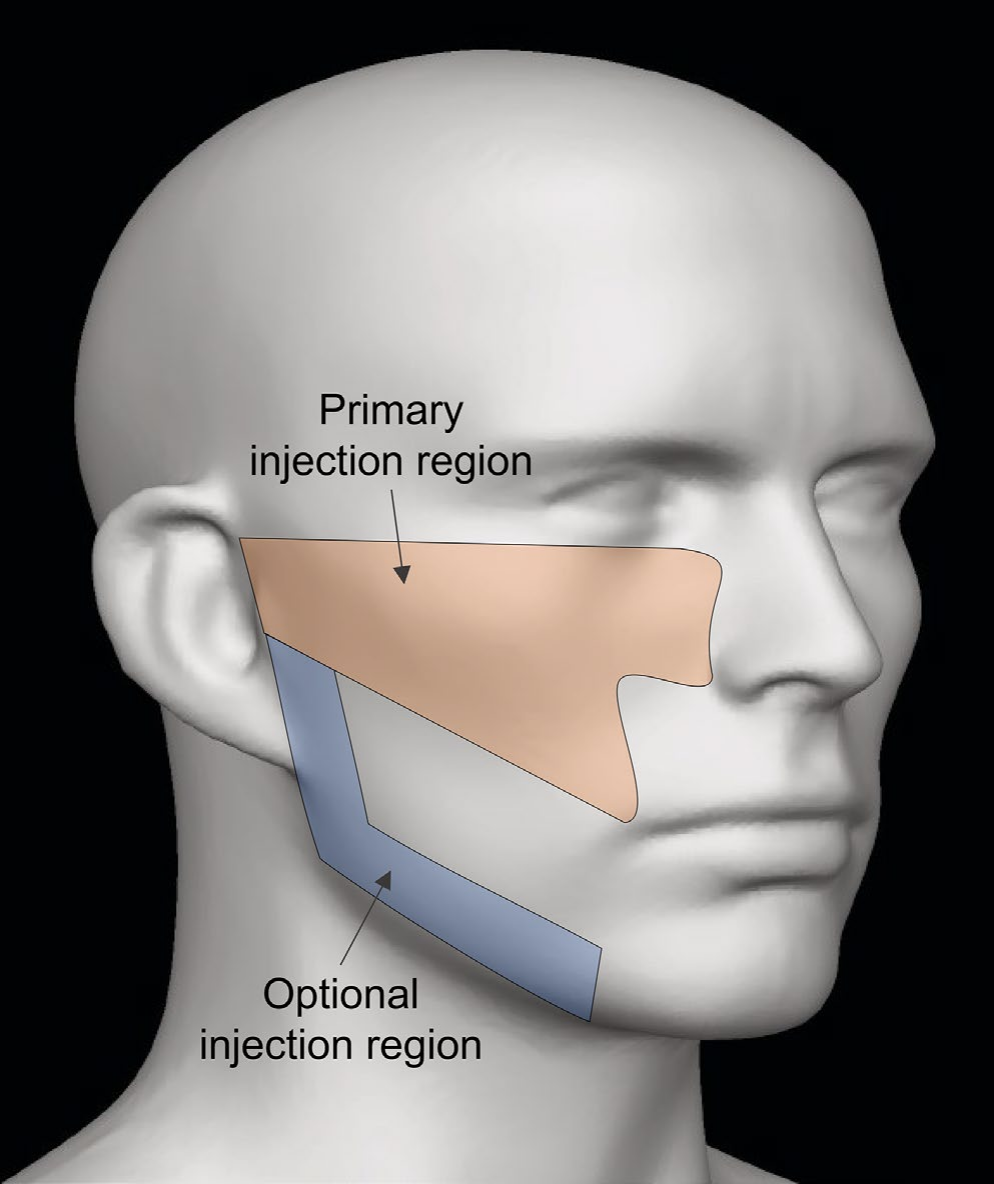
Primary and optional treatment areas.

On Day 1 (initial treatment), participants were injected with up to 6.25 mL of HA‐CaHA (20 mg/mL HA, 55.7% w/w CaHA) or HA‐CaHA‐L (20 mg/mL HA, 55.7% w/w CaHA, 0.3% lidocaine hydrochloride) in the midface and optional jawline area (Figure 1). If agreed upon by both the participant and EI, participants had the option to receive a touch‐up treatment 14 days after initial treatment (2.5 mL maximum injection volume). The injection technique was at the discretion of the TI.

### 2.4 Effectiveness

The primary effectiveness endpoint was MFVDS responder rate (i.e., the proportion of participants with an investigator‐rated ≥ 1‐grade improvement from baseline) at Month 1. Secondary endpoints were investigator‐ and participant‐rated responder rates (i.e., the percent of participants rated *Improved* or *Much improved*) on the Global Aesthetic Improvement Scale (GAIS) [14] in the midface area at Month 1 and mean improvement from baseline on the overall score on FACE‐Q Satisfaction with Cheeks and FACE‐Q Satisfaction with Facial Appearance [15, 16].

Other endpoints included responder rate on the validated Allergan Cheek Smoothness Scale (ACSS; i.e., ≥ 1‐grade improvement from baseline according to the EI) [17] and 3‐dimensional (3D) directional skin movement assessed by Vectra markerless skin tracking. With markerless skin tracking, a pair of pre‐ and post‐injection 3D images from different timepoints are aligned, the skin surfaces are tracked and mapped, and the magnitude and direction of skin movement are measured along *x*, *y*, and *z* axes. Lateral skin movement (*x* axis) is indicative of skin tightening, an upward movement (*y* axis) is suggestive of a lifting effect, and changes in the anterior direction (*z* axis) represent volumization.

### 2.5 Safety

Procedural pain (pain during injection) was assessed after each injection on an 11‐point scale ranging from 0 (no pain) to 10 (worst possible pain). The type and severity of injection site responses (ISRs) were recorded by participants in diaries for 30 days after initial and touch‐up treatments. Adverse events (AEs) were assessed by the investigator and were monitored for the duration of the study.

### 2.6 Statistical Analyses

Since the addition of lidocaine reduces treatment pain without impacting the efficacy of HA‐CaHA [18], the data from both groups are combined for all analyses. All 140 participants were included in both the modified intent‐to‐treat (mITT) and safety populations. Demographic and safety data are summarized descriptively. The responder status on the MFVDS, ACSS, and GAIS by evaluating investigator and participant were summarized with the 95% CI based on the modified mid‐p exact method for the binomial distribution. Change from baseline on the overall score on FACE‐Q Satisfaction with Cheeks, FACE‐Q Satisfaction with Facial Appearance, and directional skin movement were summarized descriptively. Additionally, for the FACE‐Q Satisfaction with Cheeks questionnaire (5 items) and FACE‐Q Satisfaction with Facial Appearance questionnaire (10 items), the sum of the responses was calculated and Rasch transformed to a score ranging from 0 (worst) to 100 (best). The Rasch transformation involves fitting raw FACE‐Q data to a probabilistic Rasch model, which converts ordinal‐level data into interval‐level measurements [19]. The resulting transformed scores offer a continuous measure reflecting the relative position of each participant's response on the continuum of satisfaction, thereby aiding interpretation.

## 3 Results

### 3.1 Participant Demographics and Baseline Disposition

The majority of participants enrolled in the study (*N* = 140; *n* = 110 for HA‐CaHA and *n* = 30 for HA‐CaHA‐L) self‐identified as female (87.1%) and White (96.4%), and the median age was 59 years (range, 30–78 years) (Table 1). The majority of participants (81.4%) had Fitzpatrick skin phototype III or IV, and the remainder (18.6%) had Fitzpatrick skin phototype I or II. Baseline MFVDS scores were predominantly grade 3 (69.3%) for all participants, including for the groups treated with HA‐CaHA (69.1%) and HA‐CaHA‐L (70.0%). Of 140 participants treated at baseline, 136 (97.1%) completed the study. The most common reason for study discontinuation was an inability to trace participants.

**TABLE 1:** Demographics and baseline characteristics.

	Total (*N* = 140)
Age group (years)	
Mean (SD)	57.2 (10.26)
Median	59
Q1, Q3	50.5, 66.0
Min, Max	30, 78
< 35	5 (3.6)
35–65	95 (67.9)
> 65	40 (28.6)
Sex, *n* (%)	
Male	18 (12.9)
Female	122 (87.1)
Fitzpatrick skin phototype	
I	1 (0.7)
II	25 (17.9)
III	73 (52.1)
IV	41 (29.3)
Baseline MFVDS	
Moderate (Grade 3)	97 (69.3)
Significant (Grade 4)	39 (27.9)
Severe (Grade 5)	4 (2.9)

*Note:* Percentages are based on the number of participants in the specified population.

### 3.2 Treatment Administration

In total, 140 participants received initial treatment, and 88 (62.9%) received the optional touch‐up treatment. Treatment was performed at the discretion of the treating investigator, allowing participants to receive injections across multiple planes and utilizing various injection techniques. Prior to initial and touch‐up treatments, 35.7% and 22.7% of participants received anesthesia, respectively. A majority of participants (60.0%) were treated with both a 27 G ½‐inch needle and a cannula, while 37.9% were treated with a cannula alone, and 2.1% were treated with a needle alone. Most participants received injections into the deep dermis (83.6% for left and right side of the face), with subdermis injections (32.9% to left side of face, 30.0% to right side of face) and “other” planes of injection (e.g., supraperiosteal; 45.7% to left side of face, 51.4% to right side of face) occurring less frequently. Fanning (80.7%) and tunneling (41.4%) were the most common injection techniques for initial treatment.

At initial treatment, the most commonly treated areas of the midface were the zygomatic arch areas in the primary injection region (Figure 1). The median injection volume was 3.50 mL for initial treatment and 1.30 mL for touch‐up treatment. Injection volumes were comparable for the left and right sides of the face.

### 3.3 Effectiveness

**FIGURE 2:**
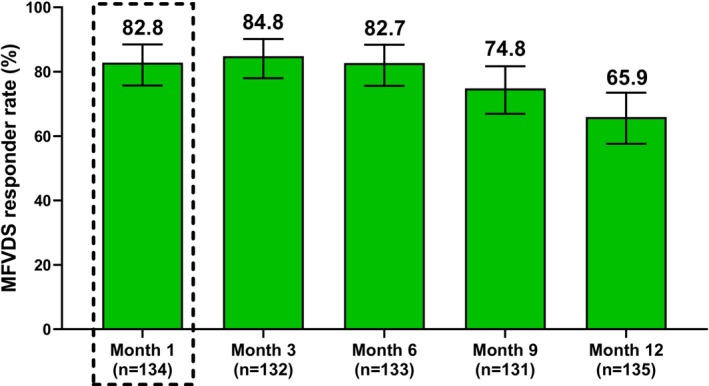
Responder rate on the Allergan Midface Volume Deficit Scale (MFVDS), defined as the percentage of participants from the combined treatment groups with ≥ 1‐grade improvement from baseline. Dashed box represents the primary effectiveness endpoint. Error bars indicate the 95% CI.

The MFVDS responder rate for the combined treatment groups based on the EI's assessment at Month 1 was 82.8% (*n* = 134; 95% CI, 75.7%–88.5%) (Figure 2). The MFVDS responder rate remained high for the duration of the study, with a responder rate of 84.8% (*n* = 132; 95% CI, 78.0%–90.2%) at Month 3, 82.7% (*n* = 133; 95% CI, 75.6%–88.4%) at Month 6, 74.8% (*n* = 131; 95% CI, 66.9%–81.7%) at Month 9, and 65.9% (*n* = 135; 95% CI, 57.6%–73.5%) at Month 12. Complete details on change from baseline on the MFVDS are provided in Table 2.

**TABLE 2:** Midface Volume Deficit Scale: Number (%) of participants by change from baseline to visit.

	Total (*N* = 140), *n* (%)
Month 1	
*N*1^a^	134
Improved 4 grades	0
Improved 3 grades	5 (3.7)
Improved 2 grades	36 (26.9)
Improved 1 grade	70 (52.2)
No change	23 (17.2)
Worsened 1 grade	0
Month 3	
*N*1^a^	132
Improved 4 grades	0
Improved 3 grades	4 (3.0)
Improved 2 grades	41 (31.1)
Improved 1 grade	67 (50.8)
No change	20 (15.2)
Worsened 1 grade	0
Month 6	
*N*1^a^	133
Improved 4 grades	0
Improved 3 grades	6 (4.5)
Improved 2 grades	33 (24.8)
Improved 1 grade	71 (53.4)
No change	23 (17.3)
Worsened 1 grade	0
Month 9	
*N*1^a^	131
Improved 4 grades	0
Improved 3 grades	1 (0.8)
Improved 2 grades	30 (22.9)
Improved 1 grade	67 (51.1)
No change	33 (25.2)
Worsened 1 grade	0
Month 12	
*N*1^a^	135
Improved 4 grades	0
Improved 3 grades	1 (0.7)
Improved 2 grades	24 (17.8)
Improved 1 grade	64 (47.4)
No change	44 (32.6)
Worsened 1 grade	2 (1.5)

^a^ The number of participants with analysis values at both baseline and specified analysis visits, used as the denominator for percentages.

**FIGURE 3:**
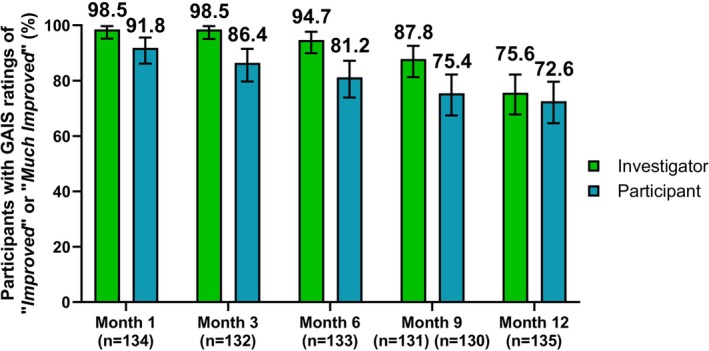
Investigator‐ and participant‐rated responder rates on the Global Aesthetic Improvement Scale (GAIS), defined as the number (%) of participants with *Improved* or *Much improved* for each assessment. Error bars indicate the 95% CI.

For the investigator‐rated GAIS, the responder rate at Months 1 and 3 was 98.5% (Month 1: 95% CI, 95.2%–99.7%; Month 3: 95% CI, 95.1%–99.7%) (Figure 3). Investigator‐assessed improvements on the GAIS remained high for the duration of the study, with responder rates of 94.7% at Month 6 (95% CI, 89.9–97.7), 87.8% at Month 9 (95% CI, 81.3–92.6) and 75.6% at Month 12 (95% CI, 67.8%–82.2%). Likewise, the responder rate for participant‐rated GAIS was 91.8% (95% CI, 86.2%–95.6%) at Month 1, 86.4% at Month 3 (95% CI, 79.7%–91.5%), 81.2% at Month 6 (95% CI, 73.9%–87.2%), 75.4% at Month 9 (95% CI, 67.4%–82.2%), and 72.6% at Month 12 (95% CI, 64.6%–79.6%).

**FIGURE 4:**
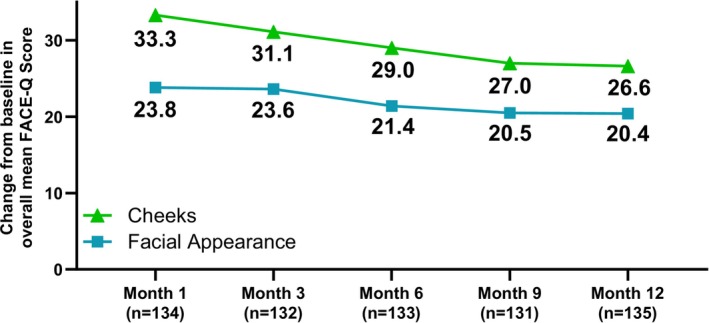
Mean change from baseline on the FACE‐Q Satisfaction with Cheeks and FACE‐Q Satisfaction with Facial Appearance questionnaires. Values represent Rasch‐transformed scores.

At baseline, the FACE‐Q Satisfaction with Cheeks overall Rasch‐transformed mean score was 34.2. By Month 1, there was a mean improvement in score of 33.3 (Figure 4). The observed improvement at Month 1 persisted through the duration of the study, with a mean improvement in score from baseline of 31.1 at Month 3, 29.0 at Month 6, 27.0 at Month 9, and 26.6 at Month 12. For the FACE‐Q Satisfaction with Facial Appearance Questionnaire, the overall Rasch‐transformed score at baseline was 34.8. By Month 1, there was a mean improvement in score of 23.8 (Figure 4). These improvements persisted through each subsequent timepoint, with a mean increase from baseline of 23.6 at Month 3, 21.4 at Month 6, 20.5 at Month 9, and 20.4 at Month 12.

**FIGURE 5:**
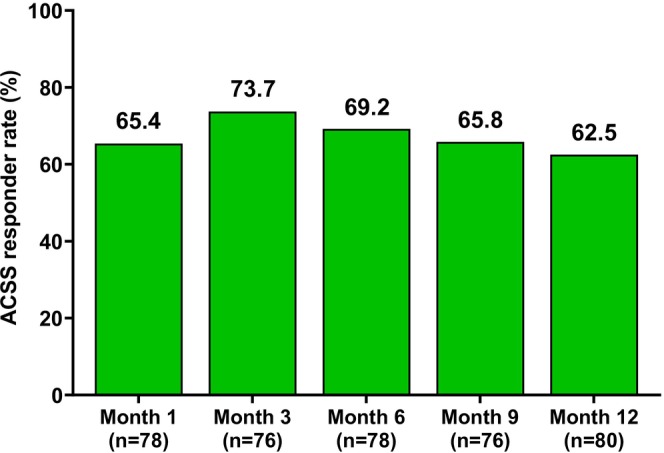
Allergan Cheek Smoothness Scale (ACSS) responder rate. The percentage of participants from the combined treatment groups meeting ACSS responder rate criteria, defined as a ≥ 1‐grade improvement from baseline. The analysis represents the subgroup of participants with moderate to severe ACSS at baseline who received injections in areas directly assessed by the ACSS (submalar and preauricular areas).

The responder rate on the ACSS at Month 1 was 65.4% (*n* = 51) for participants with moderate to severe ACSS at baseline who were injected in ACSS‐relevant areas (*n* = 80). (Figure 5). The responder rate for this subgroup of participants increased further at Months 3 (73.7%, *n* = 56) and 6 (69.2%, *n* = 54) and remained high through Months 9 (65.8%, *n* = 50) and 12 (62.5%, *n* = 50). Details on the ACSS responder rate for all treated participants are presented in Table 3.

**TABLE 3:** Allergan Cheek Smoothness Scale: Number (%) of participants with moderate to severe ACSS at baseline and ≥ 1‐grade improvement on both sides of the face from baseline by visit.

	Participants receiving injections in directly affected areas for ACSS^a^ (*n* = 80)	Others (*n* = 12)
Month 1		
*N*1^b^	78	12
Responder, *n* (%)	51 (65.4)	5 (41.7)
Month 3		
*N*1^b^	76	12
Responder, *n* (%)	56 (73.7)	7 (58.3)
Month 6		
*N*1^b^	78	11
Responder, *n* (%)	54 (69.2)	5 (45.5)
Month 9		
*N*1^b^	76	11
Responder, *n* (%)	50 (65.8)	7 (63.6)
Month 12		
*N*1^b^	80	12
Responder, *n* (%)	50 (62.5)	7 (58.3)

^a^ The directly affected areas for ACSS include the submalar and preauricular areas.

^b^ The number of participants with analysis values at both baseline and specified analysis visits, used as the denominator for percentages.

**FIGURE 6:**
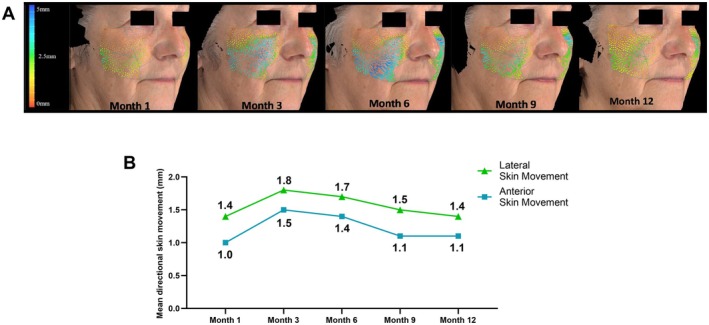
(A) Example of markerless tracking of skin movement changes over time. The color vector arrows provide an indication of the direction and magnitude of skin movement. (B) Mean directional skin movement in the lateral and anterior directions. For lateral skin movement changes, data reflect the absolute average change from baseline for the combined left (90th percentile) and right (10th percentile) midface. For anterior skin movement changes, data reflect the average change from baseline for the combined left and right midface (90th percentile).

**FIGURE 7:**
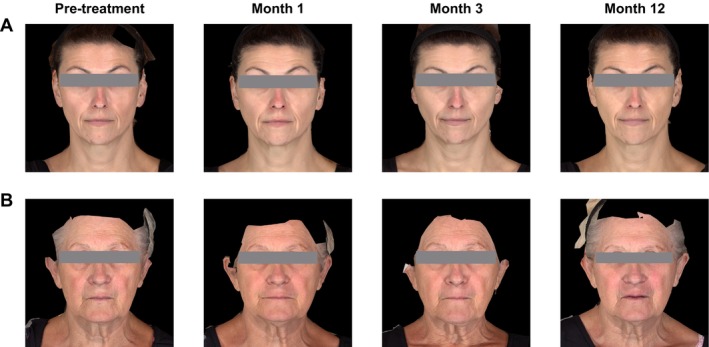
Participant photographs from baseline (pre‐treatment), Months 1, 3, and 12. (A) Participant is a white female, aged 50 years, who received a total of 2.5 mL HA‐CaHA at initial treatment and 2.2 mL at touch‐up treatment. The participant's MFVDS score was grade 3 at baseline, grade 2 at Month 1, and grade 1 at Months 3, 6, 9, and 12. (B) Participant is a white female, aged 66 years, who received a total of 3.1 mL HA‐CaHA‐L at initial treatment and 0.55 mL at touch‐up treatment. The participant's MFVDS score was grade 4 at baseline, and grade 3 at Months 1, 3, 6, 9, and 12.

Markerless tracking of the midface showed favorable directional skin movement changes from baseline in the lateral (*x*‐axis) and anterior (*z*‐axis) directions. At Month 1, the mean change from baseline in the lateral direction was 1.4 mm for the left midface and 1.5 mm for the right midface, and there was a 1‐mm change for the left and right midface in the anterior direction (Figure 6). These favorable changes sustained through 12 months, peaking at Month 3 (left midface: 1.7 mm lateral, 1.5 mm anterior; right midface: 1.8 mm lateral, 1.4 mm anterior) and Month 6 (left midface: 1.7 mm lateral, 1.4 mm anterior; right midface: 1.7 mm lateral, 1.4 mm anterior) (Figure 7). For a visual representation of skin movement at Months 6 and 12, see Videos 1, 2, 3, 4.

**VIDEO 1:** Dynamic skin movement in the midface after treatment with HA‐CaHA. The video displays midface skin movement through Month 6. Video content can be viewed at https://onlinelibrary.wiley.com/doi/10.1111/jocd.70430.

**VIDEO 2:** Dynamic skin movement in the midface after treatment with HA‐CaHA. The video displays the corresponding markerless tracking data through Month 6. Video content can be viewed at https://onlinelibrary.wiley.com/doi/10.1111/jocd.70430.

**VIDEO 3:** Dynamic skin movement in the midface after treatment with HA‐CaHA. The video displays midface skin movement through Month 12. Video content can be viewed at https://onlinelibrary.wiley.com/doi/10.1111/jocd.70430.

**VIDEO 4:** Dynamic skin movement in the midface after treatment with HA‐CaHA. The video displays the corresponding markerless tracking data through Month 12. Video content can be viewed at https://onlinelibrary.wiley.com/doi/10.1111/jocd.70430.

### 3.4 Subgroup Analyses

Subgroup analyses showed comparable effectiveness across age, sex, Fitzpatrick skin phototype, and injection volume (less than or equal to median injection volume, above median injection volume) for the primary endpoint (Table 4).

**TABLE 4:** Subgroup analyses of MFVDS responder rates at Month 1.

	Total (*N* = 140), *n*/*N*1 (%)
Baseline MFVDS	
None	0/0
Minimal	0/0
Mild	0/0
Moderate	74/92 (80.4)
Significant	35/39 (89.7)
Severe	2/3 (66.7)
Age group, years	
< 35	4/4 (100.0)
35–65	78/91 (85.7)
> 65	29/39 (74.4)
Sex	
Female	96/118 (81.4)
Male	15/16 (93.8)
Fitzpatrick skin phototype	
I/II	25/26 (96.2)
III/IV	86/108 (79.6)
V/VI	0/0
Volume injected	
≤ Median	51/65 (78.5)
> Median	60/69 (87.0)

*Note:* Percentages are calculated as 100 × (*n*/*N*1) within the specific subgroup. *n* = The number of participants with ≥ 1‐grade improvement from baseline at Month 1 in the specific subgroup category. *N*1 = The number of participants with MFVDS assessments at both baseline and Month 1 and membership in the specific subgroup category.

### 3.5 Safety

Procedural pain was minimal for the combined treatment groups, with mean scores of 4.4 after initial treatment and 3.1 after touch‐up treatment. Injection site reactions (ISRs) occurred in 95.7% of participants following initial treatment and 84.7% of participants after touch‐up treatment. The most common ISRs after initial treatment were tenderness to touch (87.0%), pain after injection (73.7%), and firmness (71.0%), and the most common ISRs after touch‐up treatment were tenderness to touch (67.1%), pain after injection (52.7%), and swelling (48.2%). Most ISRs were minimal or moderate in severity and had a duration of ≤ 2 weeks.

In total, 99 participants (70.7%) experienced treatment‐emergent AEs (TEAEs); of these, 52 (37.1%) participants had 118 events which were determined to be related to treatment. The most common treatment‐related TEAEs were injection site pain (11.4%), injection site mass (10.7%), and headache (7.1%), with all others occurring in < 5% of participants. Sixteen treatment‐related TEAEs occurring in 11 participants (7.9%) had onset ≥ 30 days posttreatment. Most treatment‐related TEAEs were mild in severity. There were no treatment‐related serious AEs. Most treatment‐related TEAEs resolved within 1 month; however, 8 mild treatment‐related TEAEs in 6 participants were ongoing at the end of the study (7 injection site masses, 1 injection site hypersensitivity).

## 4 Discussion

This open‐label, post‐marketing study showed that HA‐CaHA and HA‐CaHA‐L are effective for both immediate volumizing and a sustained lifting effect, consistent with the hybrid injectable's dual mode of action. Improvements were maintained through the 12‐month study, with peak effects at Months 3 through 6. Moreover, HA‐CaHA and HA‐CaHA‐L had favorable safety profiles that were consistent with treatment with soft‐tissue fillers.

This study showed clinically meaningful changes following treatment with HA‐CaHA and HA‐CaHA‐L in the midface across multiple qualitative and quantitative measures. Effectiveness results from MFVDS and investigator‐ and participant‐rated GAIS demonstrate the immediate volumizing effect of HA‐CaHA and HA‐CaHA‐L. By Month 1, the majority of participants (82.8%) had ≥ 1‐grade improvement on the MFVDS, with comparably high GAIS responder rates from both investigators (98.5%) and participants (91.8%) at the same timepoint. Accompanying these were substantial improvements from baseline on multiple FACE‐Q Questionnaires, including a 33‐point increase on the FACE‐Q Satisfaction with Cheeks and a 23‐point increase on the FACE‐Q Satisfaction with Facial Appearance. These findings are consistent with prior reports of rapid improvement in midface volume deficit following treatment with HA‐ and CaHA‐based fillers [20, 21, 22]. Importantly, HA‐CaHA and HA‐CaHA‐L were shown to be effective for moderate, significant, and severe midface volume deficit in both male and female participants, across multiple age groups and Fitzpatrick skin phototypes I–IV. The rapid improvements on the MFVDS, GAIS, and FACE‐Q Questionnaires were also enduring, with the majority of participants exhibiting sustained improvements through 12 months across all measures, and peak effects on the MFVDS at Month 3 (84.8% responder rate). Our findings lend further support to prior reports of high patient satisfaction and sustained aesthetic improvements following treatment with HA‐CaHA or HA‐CaHA‐L [9, 23].

More than 65% of participants reported ≥ 1‐grade improvement on the ACSS at Month 1, and this number increased through Months 3 (73.7%) and 6 (69.2%). Similar results on the ACSS were observed at Months 1, 3, and 6 in a recent study on the skin quality‐enhancing properties of the HA‐based filler VYC‐12L [17]. Vectra markerless tracking provided quantitative supporting evidence, showing the greatest changes in skin movement in both the lateral and anterior directions at Months 3 and 6, and sustained changes through 12 months. Similarly, a recent study reported improvements in dermal thickness through 120 days in a small group of participants (*n* = 15) treated once with HA‐CaHA in the unilateral midface [10]. These data are consistent with a biostimulatory effect of HA‐CaHA and HA‐CaHA‐L, demonstrating continued improvement in skin quality attributes subsequent to the initial filling effect. However, further evidence, such as histological data, is required to definitively demonstrate the biostimulatory activity of HA‐CaHA and HA‐CaHA‐L.

The safety profile of HA‐CaHA and HA‐CaHA‐L was consistent with prior studies [9, 10, 11]. Reported ISRs were of the type, severity, and duration expected with dermal filler use in soft tissues, such as pain after injection and tenderness to touch [4]. Most ISRs were minimal or moderate in severity and resolved within 2 weeks. Treatment‐related AEs included injection site pain, injection site mass, and headache; most were minimal or moderate in severity and resolved within 1 month. No participants had treatment‐related serious AEs. Taken together, HA‐CaHA was well tolerated for midface soft tissue augmentation through 12 months.

The present study provides valuable insights into effectiveness, safety, and potential skin quality improvements associated with HA‐CaHA and HA‐CaHA‐L for treating midface volume deficit. However, it is important to acknowledge certain limitations. The study's participant pool lacked diversity in Fitzpatrick Skin Phototypes, exclusively comprising individuals with Fitzpatrick skin type I/II (19%) and III/IV (81%). Additionally, while ACSS improvements and directional skin movement data suggest sustained enhancements in skin quality, these measures do not capture the full spectrum of skin quality characteristics. Including objective assessments of skin quality improvement (e.g., skin elasticity) could provide a more comprehensive evaluation of the skin quality‐improving capabilities of HA‐CaHA and HA‐CaHA‐L.

## 5 Conclusions

Treatment with HA‐CaHA/L in the midface led to an immediate and sustained increase in volume and high patient satisfaction in participants with moderate to severe midface volume deficit, with peak effects at Month 3 and 6 and results lasting up to 1 year. HA‐CaHA/L also had a favorable safety profile, similar to what is expected with other dermal fillers. These findings underscore the potential of HA‐CaHA/L as a valuable option in the portfolio of aesthetic treatments, offering both volumizing effects and promising improvements in skin quality. The dual‐action nature of HA‐CaHA/L suggests a mechanism that goes beyond traditional fillers, aligning with current demands for treatments that provide natural‐looking results [24].

## Author Contributions

Alessandro Gritti: Contributed to study execution, data collection, and interpretation. Malka Salomon: Contributed to study design, study oversight, data analysis, and interpretation. Lea Elmaleh: Contributed to study design, study oversight, data analysis, and interpretation. Graeme Kerson: Study oversight, data analysis, and interpretation. Andrew Schumacher: Contributed to study design, study oversight, data analysis, and interpretation. All authors contributed to writing, reviewing, and editing of the original draft.

## Ethics Statement

The authors confirm that the ethical policies of the journal, as noted on the journal's author guidelines page, have been adhered to. The study was conducted in accordance with the Declaration of Helsinki. All patients provided their informed consent.

## Conflicts of Interest

Alessandro Gritti: Consultant for Allergan Aesthetics, an AbbVie company. Malka Salomon: Employee of AbbVie Inc. and may own company stock. Lea Elmaleh: Employee of AbbVie Inc. and may own company stock. Graeme Kerson: Employee of AbbVie Inc. and may own company stock. Andrew Schumacher: Employee of AbbVie Inc. and may own company stock.

## Data Availability

The data that support the findings of this study are available from the corresponding author upon reasonable request.
